# Contemporary Saphenous Vein Graft Intervention: New Insights but Still More Questions

**DOI:** 10.1016/j.jscai.2024.102282

**Published:** 2024-09-26

**Authors:** Giorgio A. Medranda, Sandeep Nathan

**Affiliations:** a Division of Cardiology, Department of Medicine, NYU Langone Hospital–Long Island, Mineola, New York; b Section of Cardiology, Heart and Vascular Center, University of Chicago Medicine, Chicago, Illinois

**Keywords:** coronary artery bypass grafting, drug-eluting stents, percutaneous coronary intervention, saphenous vein graft, SCAAR

Although the use of saphenous vein grafts (SVGs) as vascular conduits during coronary artery bypass grafting (CABG) represents a time-honored approach that yet remains the de facto standard of surgical revascularization today, SVG failure rates remain high. The deterioration of SVGs, often triggering acute ischemic syndromes in the process, frequently necessitate percutaneous coronary intervention (PCI) to preserve conduit patency.^1^^,^^2^ Furthermore, this scenario often becomes an urgent and medically critical event by the invariable progression of native coronary disease in the interval between the time of the CABG surgery and the time of SVG PCI.

Despite the rapid evolution and advancements in PCI over the past 3 decades, SVG PCI remains a challenging subset associated with a higher risk of adverse procedural events including atheroembolization, no-reflow phenomenon, and perforation in the case of older bypass grafts that have become calcified. Even after successful SVG PCI, the specters of stent thrombosis, in-stent restenosis, and continued degeneration of the SVG (with need for repeat revascularization), all loom on the horizon.^1^^,^^2^ Indeed, SVG PCI is considered inherently high-risk in many contemporary definitions of PCI risk and complexity.^3^^,^^4^ As with native-vessel PCI, drug-eluting stents (DESs) appear to have a clear advantage over bare metal stents in SVG PCI but are still hampered by short-term and long-term complications.^2^ Furthermore, despite decades of data and clinical experience, much remains unknown regarding SVG PCI, including the utility of routine use of embolic protection devices (EPDs) and the long-term benefit of performing native chronic total occlusion (CTO) PCI over SVG PCI.^5^^,^^6^

A recent case report chronicling the nearly 3-decade journey of a patient with multivessel coronary artery disease and degenerated vein grafts requiring repeated high-risk interventions detailed both the scope and the complexity of the problem for such patients and ended predictably with CTO PCI of a native vessel.^7^ The majority of available literature on SVG PCI relies heavily on initial experience with early generation DES but still appears to influence contemporary practice, discouraging many from attempting SVG PCI and instead pursuing other therapeutic options. Additionally, there remains a dearth of literature regarding the outcomes of SVG PCI using contemporary DES and supported by intravascular imaging and contemporary lesion preparation techniques.

In this issue of *JSCAI*, Saidi-Seresht et al^8^ leverage the Swedish Coronary Angiography and Angioplasty Registry (SCAAR) to perform an analysis of all patients who underwent SVG PCI in Sweden between 2013 and 2020. SCAAR is a large all-comer registry recording all PCI procedures in Sweden. In 2009, SCAAR merged with other Swedish registries to create the Swedish Web-system for Enhancement and Development of Evidence-based care in Heart disease Evaluated According to Recommended Therapies (SWEDEHEART), which is funded by the Swedish government. In this analysis, the investigators studied 2198 SVG PCIs, which included 3106 contemporary DES between 2013 and 2020.^8^ The overwhelming majority of PCI (74%) were performed for acute coronary syndromes, with a substantial number of patients (∼40%) receiving >1 DES during the index procedure. Nearly half (48.5%) of PCI were performed from a transradial approach despite the high level of complexity often encountered during SVG PCI. As expected, this population represented a high-risk and chronically ill cohort of patients whose 1-year mortality was 9.2%, rising to 19.8% at 3 years with in-stent restenosis occurring frequently (10.8%). Based on an evaluation of early adverse events related to the index PCI procedure and late events related to the medical conditions of the patients being intervened upon, the authors conclude that, over the last decade, SVG PCI performed with contemporary DES was associated with high rates of long-term adverse events but overall improved stent and lesion-related outcomes.

The authors should be congratulated on their meticulous analysis of the SCAAR data, representing 1 of the largest contemporary, all-comer analyses of SVG PCI, representing over 2000 patients receiving over 3000 current-generation DES. Procedural success was high (achieved in >98% of cases) with a few complications (1.8%) with nearly half performed using a transradial approach—a practice that continues to lag in the United States for all PCI, let alone for SVG PCI. This practice and the 100% adoption of DES since 2016 underscore the progressive nature of the PCI practice in Sweden; this must be acknowledged as we strive to apply these data to US practice. The longitudinal analysis highlights the high-risk nature of this cohort, with a 1-year mortality of nearly 10% and a 3-year mortality of nearly 20%. These sobering statistics underscore the level of medical complexity that impact long-term outcomes for these patients.

Several questions remain unanswered in this complex cohort of patients. Given the origin of this data set, the cohort was expected to be homogenous and predominantly Caucasian; however, female patients were surprisingly underrepresented in this analysis, comprising just 13.8% of all subjects studied. While the reasons underlying this skew are likely multifactorial, it should be recognized that women have increased risks for both procedural and bleeding complications as well as target vessel/target lesion failure in non–SVG PCI, owing in part to their anatomical differences. Outcomes of women undergoing SVG PCI are lacking, and a subgroup analysis of female patients would shed light on the management of women with graft failure after surgery. Another area deserving attention is the use of intravascular ultrasound and EPD were not routinely reported in SCAAR. There is now ample evidence to support the use of intravascular imaging in non–SVG PCI as a means of significantly improving clinical outcomes, with differential benefit noted in complex coronary lesions.^9^^,^^10^ There is a theoretical benefit of routine adoption of intravascular ultrasound during SVG PCI to determine lesion characteristics, guide use of atheroablative therapies, and inform stent sizing and optimization. However, in the absence of large-scale contemporary data, these considerations remain speculative for this particular lesion set and must be balanced with concerns of SVG trauma and consequent complications. The combination of lower-profile intravascular imaging catheters and higher-resolution imaging might limit risk of plaque disruption (and complications) while providing valuable image information to optimize SVG PCI and further improve outcomes. Similarly, the routine use of EPD was missing from the SCARR analysis. Widespread adoption of EPD has the potential to further improve the already impressive procedural outcomes demonstrated in the Swedish experience.

Finally, the absence of index procedure-related morality and the lack of a comparator to help place the SCAAR SVG PCI outcomes into global clinical context should be noted. The absence of procedure-related mortality appears difficult to reconcile with such a complex patient population where 3-quarters of patients presented with ACS^8^ and may be related to inherent limitations of working retrospectively with a large national registry.

Future analysis of SVG PCI should address these remaining questions for women and ethnically diverse populations to help inform procedural strategy when choosing between SVG PCI and native CTO PCI. Other areas of interest include PCI outcomes of arterial grafts, non–left internal mammary artery grafts, including pedicled, free right internal mammary artery grafts or radial artery grafts. Such analyses have the potential to guide upfront selection of CABG grafts with future graft PCI in mind.

As CABG patients live longer, the importance of SVG PCI will continue to grow, as will our collective experience and our ability to apply contemporary PCI techniques to optimize outcomes. Ultimately, a robust aggregate of data, incorporating use of intravascular imaging, lesion modification tools, and EPD, is needed to demonstrate the true potential of contemporary SVG PCI, help guide therapeutic decisions for individual patients, and shape future practice guidelines applicable to this population.
